# Effects of New Zealand blackcurrant extract supplementation on muscle damage, oxidative stress, and inflammatory markers following high-intensity training in elite taekwondo athletes: a randomized, double-blind trial

**DOI:** 10.1007/s11845-026-04353-8

**Published:** 2026-03-31

**Authors:** Mustafa Kaçar, Hadi Karimkhani, Sedat Arslan

**Affiliations:** 1 Department of Nutrition and Dietetics, Istanbul Okan University, Istanbul, Turkey; 2 Department of Biochemistry, School of Medicine, Istanbul Okan University, Istanbul, Turkey; 3 Department of Nutrition and Dietetics, Bursa Uludag University, Bursa, Turkey

**Keywords:** New Zealand blackcurrant (NZBC), *Ribes Nigrum L.*, Anthocyanins, Taekwondo, Oxidative stress, Inflammation, Recovery

## Abstract

**Background:**

Exercise-induced muscle damage, oxidative stress, and inflammation can impair recovery and performance in elite athletes. Anthocyanin-rich New Zealand blackcurrant (NZBC) has been proposed as a nutritional strategy to counteract these effects.

**Methods:**

In a randomized, double-blind, placebo-controlled trial, 29 male elite taekwondo athletes received either NZBC extract (210 mg anthocyanins/day) or placebo for 7 days. Blood samples and muscle soreness ratings were collected at baseline, immediately post-exercise, 24 h, and 7 d after repeated high-intensity training sessions. Primary outcomes included creatine kinase (CK) and reactive oxygen species (ROS); secondary outcomes were total antioxidant capacity (TOAC) and inflammatory cytokines.

**Results:**

Compared with placebo, NZBC significantly attenuated the 24-h increase in CK (mean difference − 356 U/L; 95% CI − 510 to − 202) and ROS (− 1.8 units; 95% CI − 2.5 to − 1.0), while enhancing TOAC (+ 0.21 mmol Trolox equivalents; 95% CI + 0.09 to + 0.33). At 7 days, cytokine levels (IL-6, TNF-α) were lower in NZBC than placebo (IL-6: −1.2 pg/mL; 95% CI − 2.1 to − 0.3). Effect sizes were moderate to large. No adverse events were reported.

**Conclusions:**

Short-term supplementation with NZBC extract reduced exercise-induced muscle damage, oxidative stress, and inflammation in elite taekwondo athletes. These findings support the role of anthocyanin-rich NZBC as a personalized recovery aid in combat sports.

**Supplementary Information:**

The online version contains supplementary material available at 10.1007/s11845-026-04353-8.

## Introduction

Taekwondo is a high-intensity, intermittent Olympic combat sport characterized by repeated explosive efforts and frequent impact actions. Such loading patterns predispose athletes to exercise-induced muscle damage (EIMD) with consequent perturbations in sarcolemmal integrity and elevations in biochemical markers such as creatine kinase, alongside transient inflammatory and redox imbalance that, if insufficiently managed, may delay recovery and impair performance [1, 2]. Acute exercise triggers a coordinated cytokine response in skeletal muscle, notably interleukin-6 as a myokine, with downstream effects on systemic inflammation and metabolism; excessive or poorly resolved responses such as increases in tumor necrosis factor-α can exacerbate muscle soreness and prolong return to readiness [3, 4]. In parallel, reactive oxygen species generation rises and, when unmatched by endogenous antioxidant defenses, contributes to oxidative damage of lipids and proteins [1, 4].

Accordingly, recovery-oriented nutrition strategies are integral to high-performance practice. Beyond core macronutrient support, there is growing interest in plant-derived ergogenic aids whose polyphenols and anthocyanins may attenuate post-exercise oxidative and inflammatory stress and thereby support functional recovery [5–7]. New Zealand blackcurrant (Ribes nigrum L.) extracts are particularly rich in delphinidin- and cyanidin-based anthocyanins and related polyphenols with documented radical-scavenging and signaling actions [4, 7–9]. Bioavailability considerations further suggest that both absorbed anthocyanins and gut-derived microbial metabolites contribute to these effects [10].

Initial human studies indicate that New Zealand blackcurrant (NZBC) supplementation can improve exercise responses and recovery indices across modalities. In endurance and intermittent settings, NZBC has been associated with improved cycling performance and fat oxidation, and with better tolerance to high-intensity running [11–13]. Pilot work using an anthocyanin-rich NZBC extract reported reduced post-exercise oxidative stress and inflammation over several weeks [8], while studies with other anthocyanin-rich berries such as blueberries and Montmorency cherry similarly show reductions in muscle damage markers and soreness after strenuous or eccentric exercise [14, 15]. Nevertheless, randomized trials in elite combat-sport athletes remain scarce, and sport-specific evidence in taekwondo—a discipline with unique eccentric-impact demands—is limited [1, 8, 13–15].

Given this context, the present study investigated whether 7-day supplementation with a standardized NZBC extract would modulate biochemical markers of muscle damage, oxidative stress, antioxidant status, inflammatory cytokines, and perceived muscle soreness following repeated high-intensity training in elite taekwondo athletes. We hypothesized that NZBC would attenuate rises in damage and inflammatory markers while enhancing antioxidant capacity, thereby supporting more efficient recovery in this population [1, 2, 4, 7, 8, 13, 15].

## Materials and methods

### Participants

**Fig. 1:**
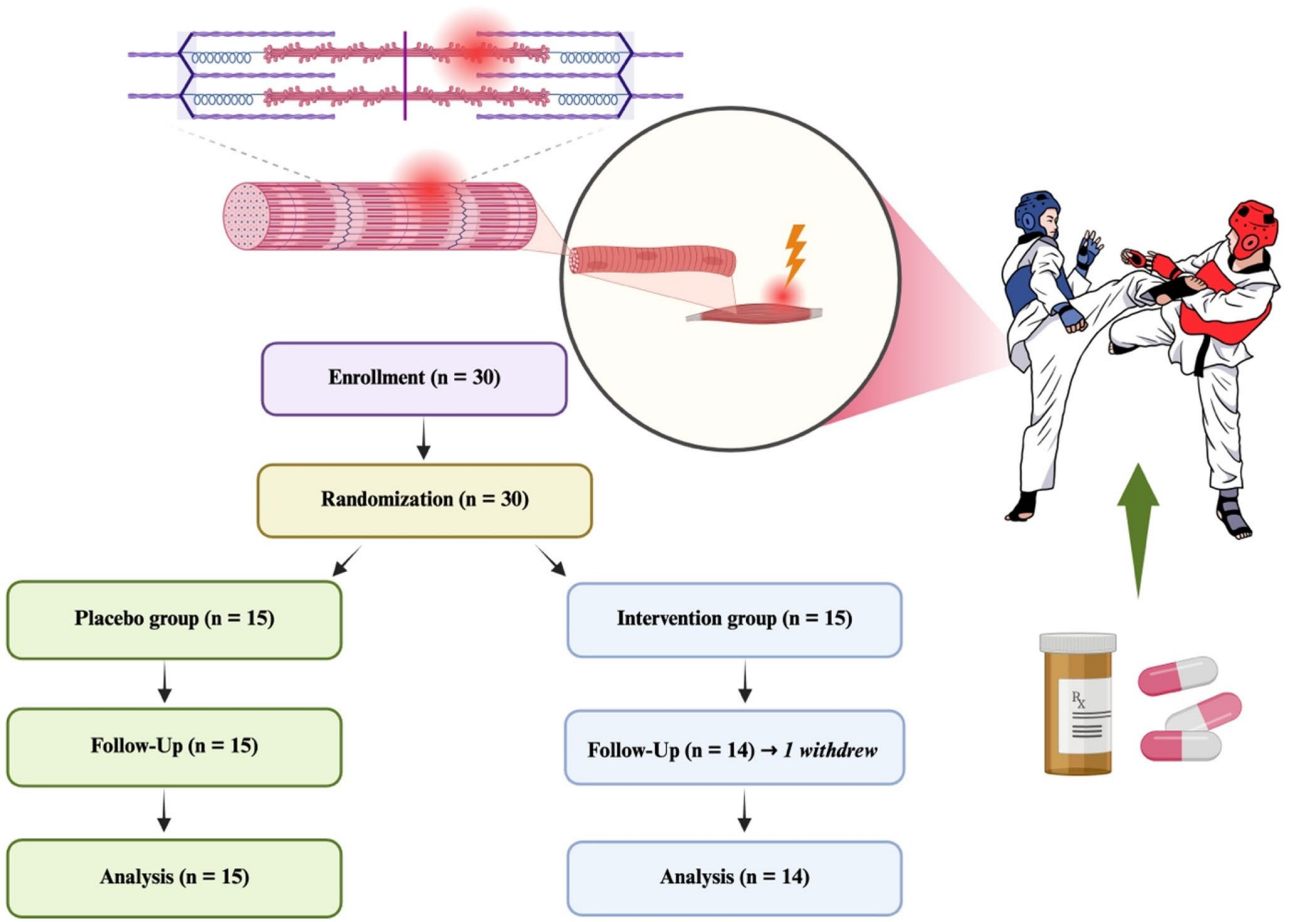
CONSORT diagram

Thirty professional male elite taekwondo athletes were initially recruited. A priori sample size estimation was performed in G*Power (version 3.1) using effect sizes from prior work (assumed f = 1.00, α = 0.05, power = 0.95), indicating 15 participants per group [16]. Athletes were randomly assigned to NZBC or placebo (1:1). One athlete in the NZBC group withdrew; thus, 14 completed NZBC and 15 placebo. Inclusion criteria were active national-level training, absence of chronic disease or injury, and no use of antioxidant/anti-inflammatory agents within four weeks; exclusion criteria were female athletes, chronic illness, or diet incompatible with the intervention. All participants provided written informed consent. Ethics approval: Biruni University Clinical Research Ethics Committee (22.12.2023, No: 2015-KAEK-80-23-33). Trial reporting follows CONSORT, and the participant flow is presented in Fig. 1 [17].

### Study design

**Fig. 2:**
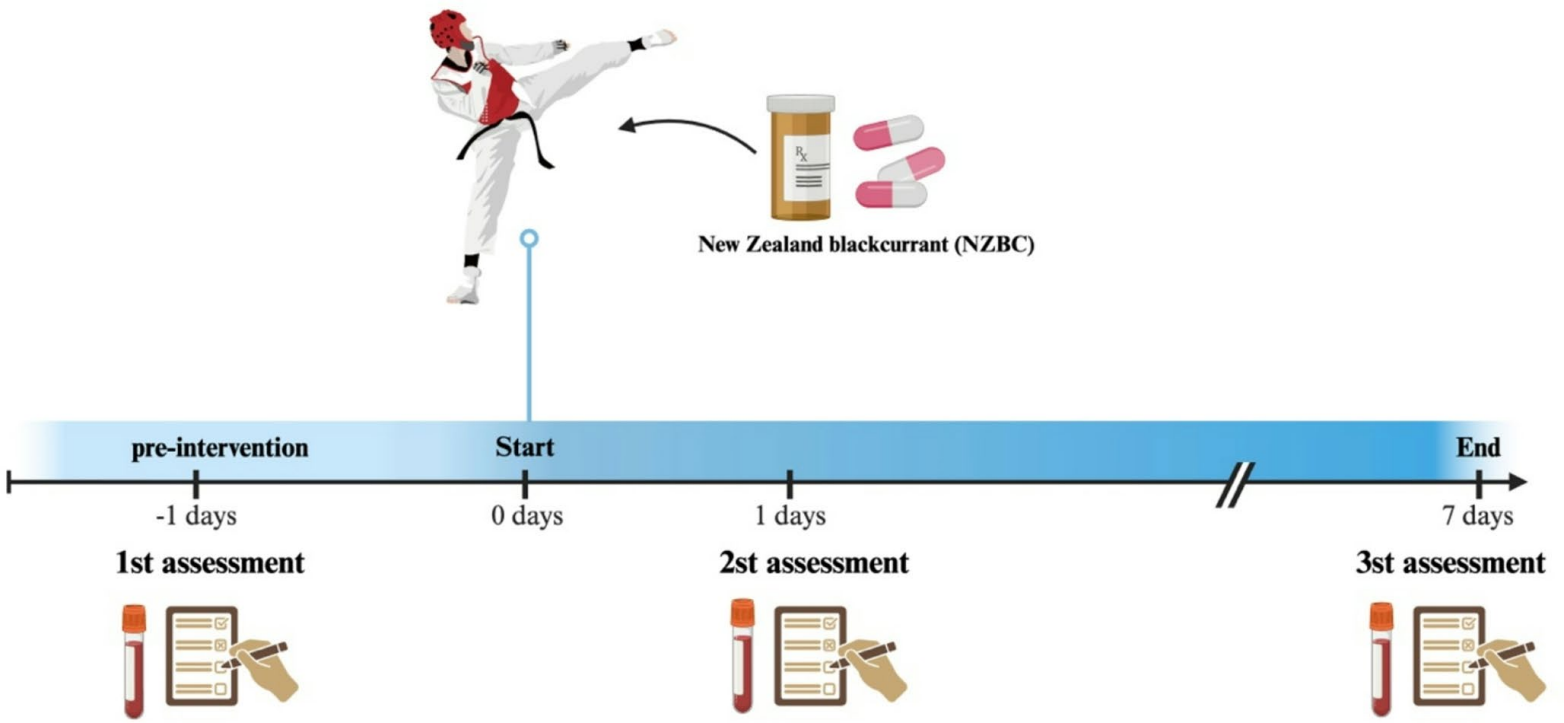
Study design

This was a double-blind, randomized, placebo-controlled trial conducted at Atik Ali Sports Club (Istanbul, Turkey) between December 2023 and June 2024. The intervention lasted 7 days, with blood sampling at baseline, 24 h, and day 7 to capture acute responses. NZBC capsules (CurraNZ^®^, Healthspan Elite, UK) were administered at 600 mg·day⁻¹ (300 mg morning, 300 mg evening), equivalent to ~ 210 mg anthocyanins·day⁻¹ per manufacturer. Placebo capsules were identical in appearance and contained maltodextrin. Participants were randomly allocated (1:1) to NZBC extract or placebo using a computer-generated random sequence prepared by an independent researcher not involved in assessments. Allocation was concealed using sequentially numbered, opaque, sealed envelopes. After baseline measurements, the study coordinator enrolled participants and assigned the next available allocation code. Participants, investigators, and outcome assessors remained blinded until completion of the analyses. The study schematic is shown in Fig. 2.

### Biochemical analysis

Plasma was separated by centrifugation and stored at − 80 °C. Plasma CK (Cat. No. 201-12-2091), protein carbonyls (201-12-5311), ROS (201-12-9433), IL-6 (201-12-0091), IL-10 (201-12-0103), TNF-α (201-12-0083), and TAC (201-12-2200) were quantified using commercial ELISA kits (Sunredbio, Shanghai, China) per manufacturer’s instructions. All assays were run in duplicate; absorbance at 450 nm was read on a microplate reader (BioTek ELx800, USA), and concentrations were derived from assay-specific standard curves (Table 1).

**Table 1:** ELISA kits and catalog numbers used in the study

ELISA test name	Catalog no
Plasma Creatine Kinase	201-12-2091
Protein Carbonyls	201-12-5311
Reactive Oxygen Species	201-12-9433
Interleukin-6	201-12-0091
Interleukin-10	201-12-0103
Tumor Necrosis Factor Alpha	201-12-0083
Total Antioxidant Capacity	201-12-2200

### Dietary and anthropometric assessments

Three-day dietary records (two training days, one weekend day) were analyzed using the BeBiS Nutrition Information System (version 8.2; Mavi Elma Yazılım, Istanbul, Turkey) [18]. Anthropometrics (height, weight, waist, chest, shoulder circumferences) were obtained by trained staff using standardized protocols.

### ELISA analyses

**Fig. 3:**
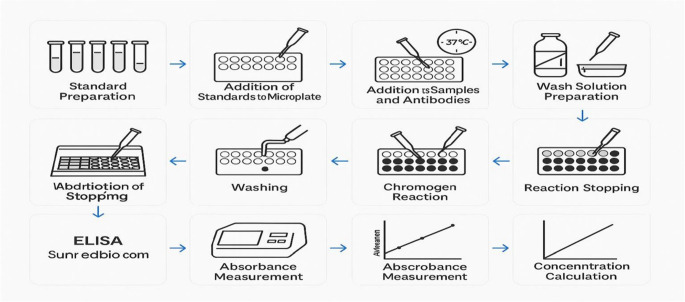
ELISA test protocol

Plasma concentrations of creatine kinase, protein carbonyls, reactive oxygen species, IL-6, IL-10, TNF-α, and total antioxidant capacity were measured using commercial ELISA kits (Sunredbio, Shanghai, China; catalog numbers in Supplementary Table 1). All assays were performed in duplicate according to the manufacturer’s instructions (Fig. 3). Absorbance was read at 450 nm using a microplate reader (BioTek ELx800, USA), and concentrations were calculated from standard curves generated for each assay.

#### Step 1: Preparation of standard solutions

Standard solution was prepared in six test tubes by serial dilution method. In the first tube, 120 µL of the original standard solution and 120 µL of standard diluent were added and homogenized on a magnetic stirrer. 120 µL of the mixture was transferred to the second tube with 120 µL of diluent. This process was continued up to six tubes while maintaining the same ratio. Thus, equally decreasing standard solutions were obtained.

#### Step 2: Adding standards on microtiter plate

Of the standard solutions, 50 µL of the original standard was added to the first well and 50 µL of the diluted standard solutions were added to the other six wells, respectively.

#### Step 3: Adding sample and antibodies

To each well (except standard wells), 10 µL of antibody solution and 40 µL of plasma sample were added. Then 50 µL HRP (horseradish peroxidase) conjugate was added to all wells.

#### Step 4: Incubation

The microtiter plate was sealed with adhesive strip and incubated at 37 °C for 60 min with gentle shaking.

#### Step 5: Preparation of the wash solution

The washing solution was prepared by diluting 1:30 with distilled water (e.g., 20 µL washing buffer + 580 µL distilled water).

#### Step 6: Washing process

All wells were washed five times using the ELISA washing device.

#### Step 7: Chromogen reaction

To each well, 50 µL of Chromogen Solution A was added followed by 50 µL of Chromogen Solution B. The plate was gently shaken and incubated at 37 °C for 10 min, protected from light.

#### Step 8: Stopping the reaction

The reaction was terminated by adding 50 µL of stop solution to each well. The blue color changed to yellow and the reaction was observed to be complete.

#### Step 9: Absorbance measurement

Following the stop, the ELISA was placed in the ELISA reader for measurement at a wavelength of 450 nm within a maximum of 5 min. The empty well was used as the zero value.

#### Step 10: Concentration calculation

A standard curve was constructed with absorbance values corresponding to the standards. The concentrations corresponding to the sample values were calculated using a linear regression equation.

### Statistical Analysis

Analyses were performed in R (version 4.3.1; R Foundation for Statistical Computing, Vienna, Austria) [19]. Data normality was assessed with the Shapiro–Wilk test [20], and homogeneity of variances with Levene’s test [21]. Baseline between-group comparisons used independent t-tests or Mann–Whitney U as appropriate. Longitudinal effects were evaluated using repeated-measures ANOVA with group × time; when sphericity was violated, Greenhouse–Geisser corrections were applied [22]. As a sensitivity analysis, linear mixed-effects models were fitted using the lme4 package to account for within-subject clustering [23]. Post hoc pairwise tests used Bonferroni-adjusted p-values [24]. Effect sizes were reported alongside p (Cohen’s *d* for pairwise; partial η² for ANOVA). Unless stated otherwise, data are mean ± SD, and α = 0.05 (two-tailed).

## Results

Of the 30 elite male taekwondo athletes initially randomized, 29 completed the study, as one participant in the NZBC group withdrew before the post-baseline assessment. Therefore, the final intention-to-treat (ITT) analysis included *n* = 29 (NZBC, *n* = 14; placebo, *n* = 15). Groups were comparable at baseline with respect to age, anthropometric variables (height, weight, BMI, waist, chest, and shoulder circumferences), and duration of professional taekwondo practice (all *p* > 0.05). None of the participants reported alcohol or tobacco use, and all were full-time athletes. Baseline characteristics are summarized in Table 2. Dietary intake did not differ between groups during the intervention (energy and macronutrients; Supplementary Table S1).

**Table 2:** Baseline characteristics of participants

	Placebo (*n* = 15)		NZBC (*n* = 14)		Total (*N* = 29)		*p*
	Median (Lower- Upper)	x̄±s	Median (Lower- Upper)	x̄±s	Median (Lower-Upper)	µ ± σ	
Age	24 (19–28)	23.6 ± 2.82	24 (20–28)	24.36 ± 2.68	24 (19–28)	23.97 ± 2.73	0.466_t_
Height (cm)	176 (160–190)	175.73 ± 7.87	176 (160–190)	177.07 ± 8.48	177 (160–190)	176.38 ± 8.05	0.663_t_
Weight (kg)	75 (57–110)	77.07 ± 14.47	75 (56–105)	79.14 ± 14.59	77 (56–110)	78.07 ± 14.31	0.704_t_
Waist circumference (cm)	84 (76–95)	85.13 ± 5.68	84 (75–95)	86.36 ± 5.89	86 (75–95)	85.72 ± 5.71	0.574_t_
Shoulder width (cm)	109 (93–126)	109.2 ± 11.08	109 (92–125)	109.29 ± 10.84	109 (92–126)	109.24 ± 10.77	0.983_z_
Chest circumference (cm)	95 (86–105)	95.87 ± 6.6	95 (84–110)	94.79 ± 7.34	95 (84–110)	95.34 ± 6.86	0.680_t_
BMI (kg/m^2^)	23.77 (21.45–32.14)	24.75 ± 2.84	23.77 (21.34–29.71)	25.03 ± 2.77	23.85 (21.34–32.14)	24.89 ± 2.76	0.791_t_
Duration of Professional Taekwondo (Years)	12 (8–15)	12.07 ± 2.34	12 (10–16)	12.93 ± 2.43	12 (8–16)	12.48 ± 2.38	0.318_z_

t: Independent samples t test; Mann Whitney U test; :*p* < 0,05

Group×time interaction for VAS approached significance only on day 3 at 12 h post-training, favoring NZBC (t = − 2.691, *p* = 0.012). No between-group differences were detected at baseline or 24 h time points (all *p* > 0.05; Table 3). Given the ordinal nature of VAS and repeated measures, these contrasts should be interpreted cautiously.

**Table 3:** Muscle soreness (VAS) across training days

	Placebo (*n* = 15)		NZBC (*n* = 14)		Total (*N* = 29)		*p*
	Median	x̄±s	Median	x̄±s	Median	µ ± σ	
	(Lower-Upper)		(Lower-Upper)		(Lower-Upper)		
Baseline VAS score	0	0 ± 0	0	0 ± 0	0	0 ± 0	1.000z
	(0–0)		(0–0)		(0–0)		
12 h post-first training VAS score	3	2.47 ± 1.6	3	2.57 ± 1.4	3	2.52 ± 1.48	0.853t
	(0–5)		(0–5)		(0–5)		
24 h post-first training VAS score	0	0.87 ± 1.3	0	0.36 ± 0.63	0	0.62 ± 1.05	0.366z
	(0–4)		(0–2)		(0–4)		
Baseline VAS score on the second training day	0	0.07 ± 0.26	0	0.29 ± 1.07	0	0.17 ± 0.76	0.960z
	(0–1)		(0–4)		(0–4)		
12 h post-second training VAS score	3	2.93 ± 1.79	3	2.21 ± 1.25	3	2.59 ± 1.57	0.176z
	(0–5)		(1–5)		(0–5)		
24 h post-second training VAS score	1	0.8 ± 0.86	1	0.36 ± 0.63	0	0.59 ± 0.78	0.111z
	(0–3)		(0–2)		(0–3)		
Baseline VAS score on the third training day	0	0.07 ± 0.26	0	0 ± 0	0	0.03 ± 0.19	0.370z
	(0–1)		(0–0)		(0–1)		
12 h post-third training VAS score	3	3.47 ± 0.92	3	2.5 ± 1.02	3	3.00 ± 1.07	0.012*t
	(2–5)		(1–4)		(1–5)		
24 h post-third training VAS score	1	0.60 ± 0.63	1	0.36 ± 0.50	0	0.48 ± 0.57	0.263t
	(0–2)		(0–1)		(0–2)		

t: Independent samples t test; z: Mann Whitney U test; *:*p* < 0.05

Creatine kinase (CK) demonstrated a significant group × time effect (F = 9.724, *p* = 0.004; overall trajectory F = 4.902, *p* = 0.016). At 24 h post-training, the increase was substantially greater in the placebo group (Δ = 56.5 U/L; 95% CI: 34.2–78.8) compared with NZBC (Δ = 13.5 U/L; 95% CI: −2.1–29.1). By day 7, CK levels had largely returned toward baseline, with smaller changes observed in both groups (placebo Δ = 14.8 U/L; 95% CI: −5.0–34.6; NZBC Δ = 5.1 U/L; 95% CI: −12.4–22.6).

Reactive oxygen species (ROS) displayed robust between-group differences across all time points (F = 67.781, *p* < 0.001). ROS increased markedly in placebo (Δ = 1241 U/L; 95% CI: 890–1592 at 24 h), whereas NZBC supplementation reduced ROS (Δ = −546 U/L; 95% CI: −1120 to 28 at 24 h). By day 7, the placebo group remained elevated (Δ = 567 U/L; 95% CI: 210–924), while NZBC showed further reduction (Δ = −782 U/L; 95% CI: −1480 to − 84).

**Fig. 4:**
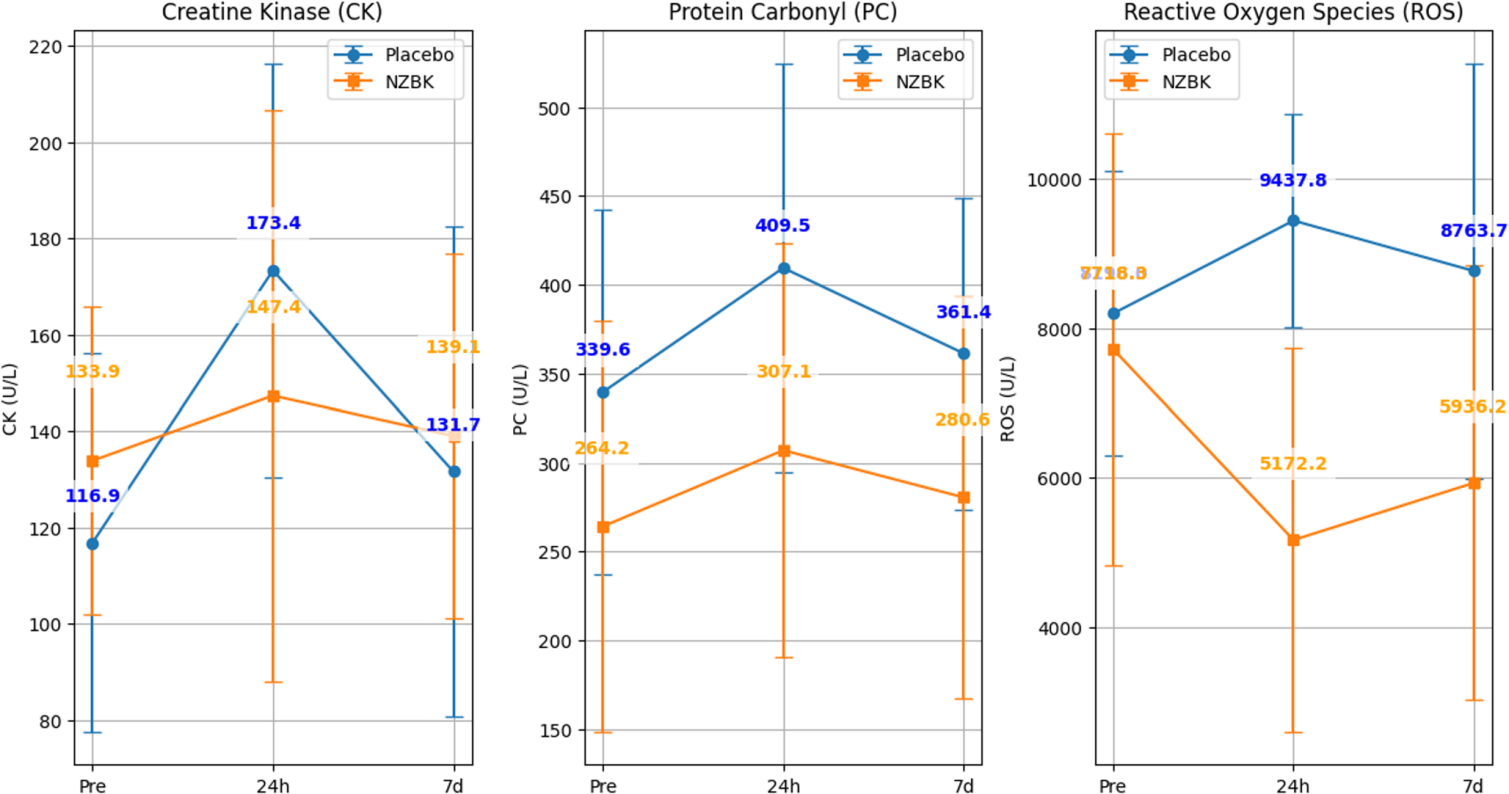
CK, ROS, and protein carbonyls over time (baseline, 24 h, day 7) in NZBC vs. placebo. Points are mean ± SEM. p values from linear mixed-effects models (random intercept for participant)

Protein carbonyls (PC) showed no significant group × time interaction (*p* > 0.05), indicating that oxidative protein damage was not influenced by the intervention. Longitudinal patterns of CK, ROS, and PC are presented in Fig. 4.

**Fig. 5:**
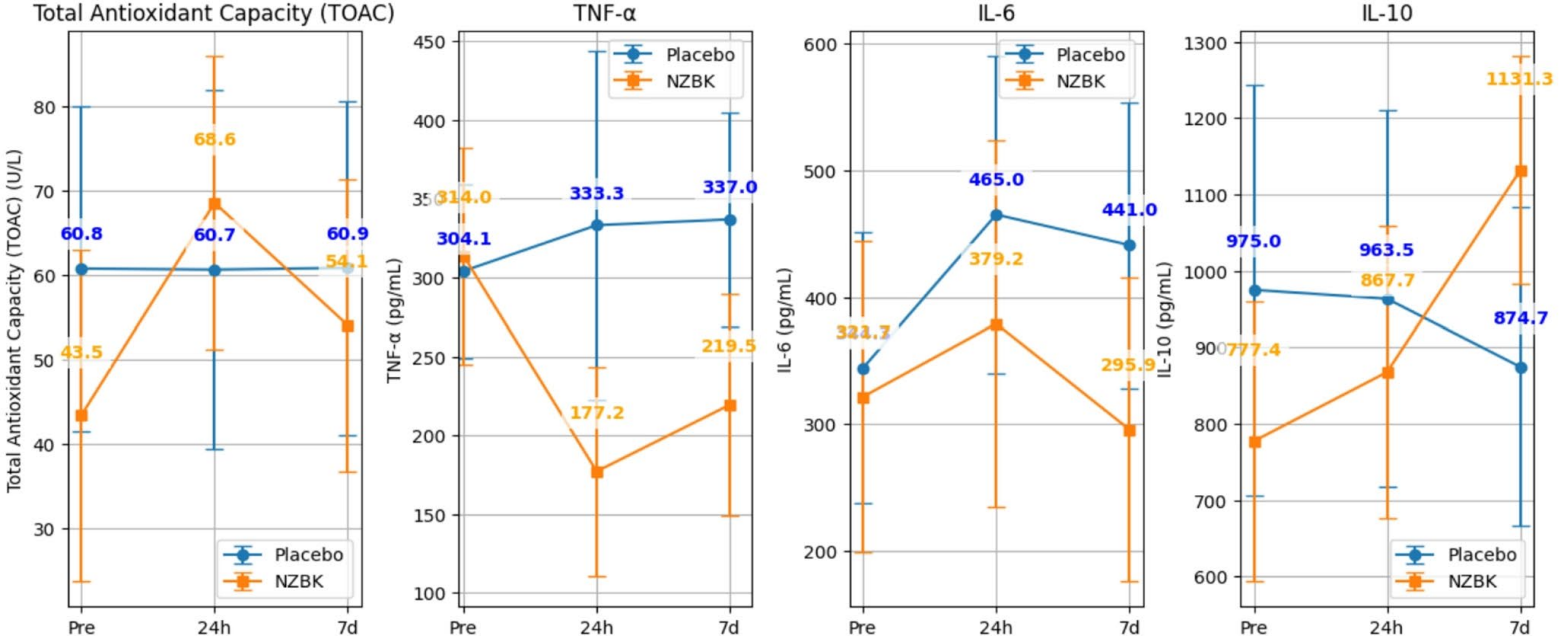
TOAC, TNF-α, IL-6, and IL-10 over time. Same analysis and display conventions as Fig. 4

Total antioxidant capacity (TOAC) increased significantly in the NZBC group relative to placebo at all time points (F = 76.870, *p* < 0.001), reflecting enhanced systemic antioxidant defenses. Pro-inflammatory cytokines showed consistent divergence: TNF-α rose in placebo but declined in NZBC (F = 40.848, *p* < 0.001), while IL-6 increased in placebo and decreased in NZBC (F = 11.734, *p* = 0.001). Conversely, IL-10 levels were higher in NZBC at all time points, particularly at 24 h, indicating an acute anti-inflammatory effect (F = 23.176, *p* < 0.001). These trajectories are illustrated in Fig. 5.

**Fig. 6:**
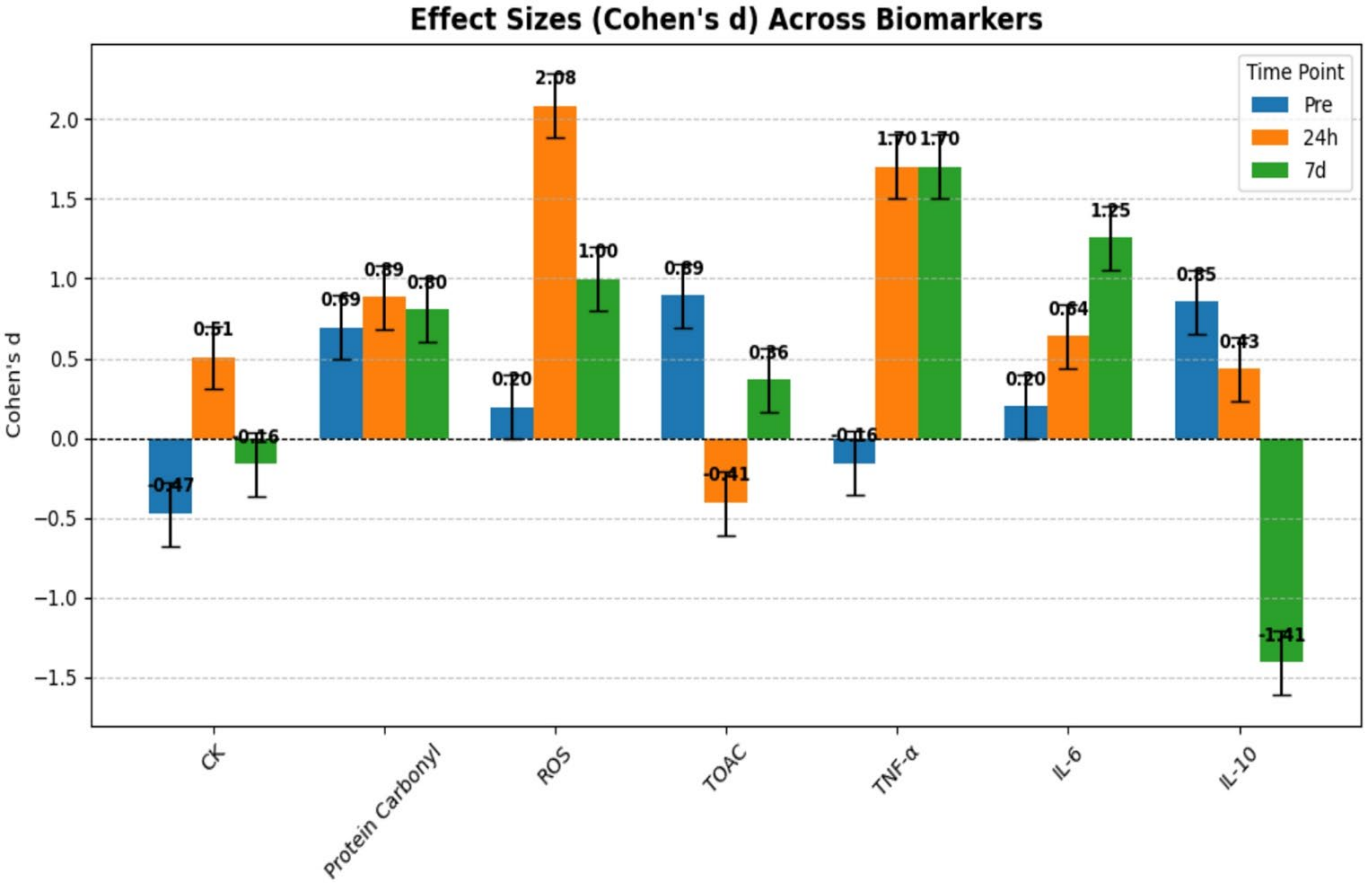
Cohen’s d values of the findings obtained by ELISA method are given. (d < 0.5; weak effect, 0.5 d < < 0.8; medium- sized effect, d > 0.8; strong effect)

Effect size calculations using Cohen’s d supported the clinical relevance of the findings. Strong effects (d > 0.8) were observed for TNF-α and protein carbonyls, while IL-6 and ROS showed large to very large effects (up to d = 2.08). Moderate effects were observed for CK and TOAC, and variable but meaningful effects for IL-10. Importantly, CK variability was lower in the NZBC group, suggesting greater stability in muscle damage markers. Collectively, these analyses reinforce that short-term NZBC supplementation reduced exercise-induced muscle damage, attenuated oxidative stress, and favorably modulated inflammatory responses in elite taekwondo athletes (Fig. 6).

## Discussion

This randomized, double-blind, placebo-controlled trial in elite taekwondo athletes showed that 7-day New Zealand blackcurrant (NZBC) supplementation tempered exercise-induced disturbances—smaller rises in creatine kinase (CK) and reactive oxygen species (ROS), higher total antioxidant capacity (TAC), and a more favorable cytokine profile (lower TNF-α and IL-6 with a transient IL-10 rise)—relative to placebo. Group×time analyses confirmed these effects for CK and TAC and demonstrated a significant IL-10 increase confined to NZBC, while IL-6 rose at 24 h only in placebo and TNF-α was lower at 24 h in NZBC. These observations align with contemporary syntheses showing that anthocyanin-rich strategies, including NZBC, can modulate (rather than abolish) redox-inflammatory signaling and support recovery in athletes [25–27].

Our athletes exhibited a smaller CK rise and quicker normalization with NZBC. Recent quantitative reviews report reduced CK and soreness with anthocyanin-rich foods, with effects emerging from ~ 24–48 h post-exercise and scaling (weakly) with dose [28]. Focusing on NZBC specifically, a 2020 meta-analysis found a small but significant performance benefit with effective anthocyanin doses ~ 105–210 mg [25]—a range matched by our protocol (210 mg·day⁻¹ across 7 days). The convergence between our damage trajectory and these performance data suggests membrane-stabilizing and secondary-damage-limiting actions that are especially relevant for taekwondo’s eccentric/impact loading [25, 26].

NZBC blunted the acute ROS increase and elevated TAC versus placebo. Contemporary meta-analytic and narrative work indicates berry anthocyanins enhance antioxidant capacity and tune redox-sensitive pathways without over-quenching adaptive signals [26, 28, 29]. Mechanistically, cross-talk between Nrf2 and NF-κB provides a plausible axis for simultaneous antioxidant up-regulation and inflammatory restraint [30]. Protein carbonyls trended lower without reaching significance—consistent with variability and with umbrella-review evidence that not all oxidative endpoints respond uniformly across short protocols [31].

We observed a placebo-only IL-6 rise at 24 h and lower TNF-α with NZBC at 24 h, alongside a transient IL-10 increase restricted to NZBC, indicating a “nudge-not-block” immunomodulation conducive to recovery. This pattern agrees with recent anthocyanin syntheses reporting moderated pro-inflammatory signaling and occasional IL-10 facilitation [26, 28].

Our split dosing (AM/PM) for 7 days delivered 210 mg anthocyanins·day⁻¹—a regimen mirrored in newer NZBC studies exploring repeat dosing and responder heterogeneity [32]. Inter-individual variability likely reflects baseline metabolic function and gut-microbiome-derived metabolite profiles; very recent trials link specific phenolic metabolites to vascular/inflammatory shifts after blackcurrant intake [29, 33]. Future work in combat sports should compare pre-exercise priming vs. recovery-focused dosing, and consider co-ingestion with carbohydrate or protein [26, 29].

Although we did not include performance endpoints, NZBC has shown benefits for high-intensity intermittent running and other performance tasks in controlled studies, particularly with 7-day loading at ~ 105–315 mg anthocyanins [25, 26]. Given our biomarker shifts (CK/ROS/TAC/cytokines), sport-specific performance testing in taekwondo (e.g., repeated-kick and match-simulation protocols) is warranted to determine functional translation.

Not all polyphenol interventions improve all outcomes—e.g., a 2024 chokeberry-containing juice showed no group effect on CK or cytokines during a 6-day HIIT protocol, likely due to dose/composition and timing relative to sampling [31, 34]. Our use of a standardized NZBC extract with defined anthocyanin content and a 7-day run-in may partly explain the clearer signal in redox-inflammatory markers.

From a broader perspective, recovery-oriented nutritional strategies that modulate physiological stress and inflammation may have downstream relevance to non-communicable disease risk and, consequently, healthcare expenditure [35]. In sum, our findings extend recent evidence that NZBC can beneficially tune redox-inflammatory responses after intensive training, with biologically plausible mechanisms (Nrf2↔NF-κB cross-talk) and dosing that matches effective ranges in the literature [25–27, 29].

### Limitations

This trial had a modest sample and a short, 7-day exposure, limiting inference on chronic adaptations. The male-only cohort constrains generalizability to female athletes. Although diet was recorded, residual nutritional confounding is possible. Finally, without sport-specific performance endpoints, the translation from biochemical changes to functional recovery remains inferential. Future multi-center studies with longer supplementation, inclusion of female athletes, and integration of taekwondo-relevant performance tests and mechanistic probes (e.g., NF-κB/Nrf2 targets, endothelial function) are warranted [26, 27].

### Conclusions

In conclusion, this study provides novel evidence that short-term supplementation with New Zealand blackcurrant extract can attenuate biochemical markers of muscle damage, oxidative stress, and inflammation in elite taekwondo athletes undergoing repeated high-intensity training. By reducing CK and ROS, enhancing antioxidant capacity, and modulating cytokine responses, NZBC supplementation appears to support more efficient recovery and maintenance of physiological homeostasis during intensive exercise.

These results strengthen the rationale for incorporating anthocyanin-rich supplements into recovery strategies for combat sport athletes. The findings not only confirm the biological plausibility of anthocyanin-mediated protection but also highlight the practical potential of NZBC as a safe, well-tolerated, and easily applicable nutritional intervention.

Future research should focus on longer-term interventions, the inclusion of both sexes, and direct assessments of athletic performance outcomes to fully establish the translational value of these findings. If confirmed, NZBC supplementation could represent a valuable addition to evidence-based nutritional practices aimed at optimizing recovery, reducing injury risk, and enhancing readiness in high-performance sport.

## Supplementary Information

Below is the link to the electronic supplementary material.

Supplementary Material 1
